# Tension pneumothorax from large bowel herniation and perforation as a late presentation of traumatic diaphragmatic hernia during pregnancy: a case report

**DOI:** 10.1186/s12245-025-00843-1

**Published:** 2025-03-03

**Authors:** Ákos Sóti, Gábor Nagy, Zoltán Győri, Tamás Vass, László Hetzman, Bánk Gábor Fenyves, Csaba Varga

**Affiliations:** 1 Department of Emergency Medicine, Semmelweis University, Üllői u. 78/A, Budapest, H-1082 Hungary; 2 Medical Imaging Centre, Semmelweis University, Korányi Sándor u. 2., Budapest, H-1083 Hungary; 3 Department of Surgery, Transplantation and Gastroenterology, Semmelweis University, Üllői u. 78., Budapest, H-1082 Hungary

**Keywords:** Diaphragmatic hernia, Large bowel perforation, Empyema, Tension pneumothorax, Pregnancy, Dyspnea

## Abstract

**Background:**

Diaphragmatic hernias can be congenital or acquired, with trauma being the primary cause of the latter. Both types may have delayed presentations, with abdominal organs protruding into the thoracic cavity, causing symptoms of varying severity. Pregnancy can sometimes precipitate the condition. Tension pneumothorax resulting from bowel perforation into the thorax is exceptionally rare, with only a few cases reported. To the best of the authors knowledge, this is the third documented case of a late-presenting trauma-related diaphragmatic hernia during pregnancy, complicated by tension pneumothorax.

**Case presentation:**

A 30-year-old woman, 29 weeks pregnant, was referred to Semmelweis University emergency department with moderate dyspnea. Initial investigation revealed tension pneumothorax. Chest tube placement released air, pus, and feces. Computer tomography identified a diaphragmatic hernia with bowel incarceration and perforation as the underlying cause. The patient underwent a delayed cesarean section and surgical repair, with a good outcome. A history of thoracic trauma eight years prior was later revealed.

**Conclusion:**

Evaluating pregnant patients with shortness of breath in the emergency department is challenging. Identifying a history of thoracic or abdominal trauma is crucial, as this can raise the suspicion of diaphragmatic hernia, which can present with a wide range of symptoms. Spontaneous tension pneumothorax in pregnant women is extremely rare and requires cautious management. A multidisciplinary approach is crucial for the successful treatment of maternal diaphragmatic hernia.

**Supplementary Information:**

The online version contains supplementary material available at 10.1186/s12245-025-00843-1.

## Background

Diaphragmatic hernia (DH) is a rare, potentially life-threatening defect in the diaphragm that allows abdominal organs to protrude into the thoracic cavity. It can be congenital or acquired. Congenital diaphragmatic hernia (CDH) is typically diagnosed and treated during the perinatal period but may exceptionally manifest in adulthood, often triggered by increased intra-abdominal pressure, such as during pregnancy [1, 2]. Acquired diaphragmatic hernia (ADH), which is caused primarily by trauma or hepatic surgery, has a low incidence (0.46%) but a high mortality rate (8.8–19.8%) [3, 4]. Traumatic diaphragm injuries often remain undetected initially, with symptoms and complications occurring only months or years later [5, 6]. Severity varies widely depending on the herniated organs and resulting complications, such as bowel or gastric herniation, volvulus and incarceration. Associated tension pneumothorax with or without confirmed bowel perforation is less frequent – especially with late presentation in trauma - and has been reported in only a few cases [7–10]. 

Maternal diaphragmatic hernia (MDH) during pregnancy is rare. A systematic review identified only 158 cases, 34 of which were trauma related [11]. Another review of 69 cases found trauma in 25% [12]. Pneumothorax (PTX), especially tension pneumothorax, has been reported in isolated cases of trauma-related MDH. The coexistence of a late-presenting trauma-related diaphragmatic hernia, bowel perforation, and tension pneumothorax during pregnancy is exceptionally rare [13–15]. 

### Case presentation

A 30-year-old woman, 29 weeks pregnant, was referred to Semmelweis University emergency department with moderate dyspnea. Her symptoms began ten days earlier with stomach pain, chills, and vomiting. Initially diagnosed with gastritis (see laboratory findings in Table 1.), she returned to the local emergency department on the day of admission with the complaint of “shortness of breathing, left side pain and uncomfortable feeling at peeing”. Abdominal and obstetric ultrasounds were normal, but fetal tachycardia (170/min) was noted. Laboratory tests revealed elevated inflammatory markers and a positive urinary test. (Table 1.) She was treated with intravenous antibiotics (ampicillin 1 g), metamizole 1 g, drotaverine 40 mg, bencyclan 50 mg and crystalloid infusion and referred to Semmelweis University obstetric department with a diagnosis of pyelonephritis. Her dyspnea worsened upon arrival, though fetal assessment was normal. She was then referred to the emergency department for further evaluation.

**Table 1:** Laboratory findings

		initial findings (10 days before admission)	on admission	reference range
WBC (G/l)		17.3	26.4	4.0–10.0
Neutrophils (%)		91.2	89.1	45.0–70.0%
CRP (mg/L)		10.1	308.9	< 10.0
Procalcitonin (ng/L)		-	8.19	< 0,5
D-dimer (mg/L)		-	2.34	< 0.5
Urinary test	blood	-	+++	-
	protein	-	+++	-
	leukocytes	-	++	-

Upon admission, the patient reported left-sided, movement-dependent chest pain and worsening shortness of breath. Physical examination revealed decreased air entry on the left side and dull percussion over the lung base. Mild tachycardia, tachypnoea and oxygen demand was found (Table 2.) No other abnormal findings were noted.

**Table 2:** Vital signs

respiratory rate	30/min
oxygen saturation	92% - > 98% on 4 L/min oxygen
heart rate	124/min
blood pressure	124/83 mmHg
capillary refill time	2 s
Glasgow Coma Scale	15
temperature	36.8 °C.

Arterial blood gas analysis revealed mild alkalosis, hypoxia, hypocapnia, and elevated lactate (Table 3.)

**Table 3:** Blood gas analysis on admission

FiO_2_	0,21	
pH	7.444	
pCO_2_	25.9	mmHg
pO_2_	66.6	mmHg
HCO_3_	17.8	mmol/L
Base Excess	-4.7	mmol/L
lactate	2.9	mmol/L

A bedside chest ultrasound suggested possible left lung base consolidation but was otherwise inconclusive. She was treated with intravenous paracetamol (1 g) and oxygen therapy.

**Fig. 1:**
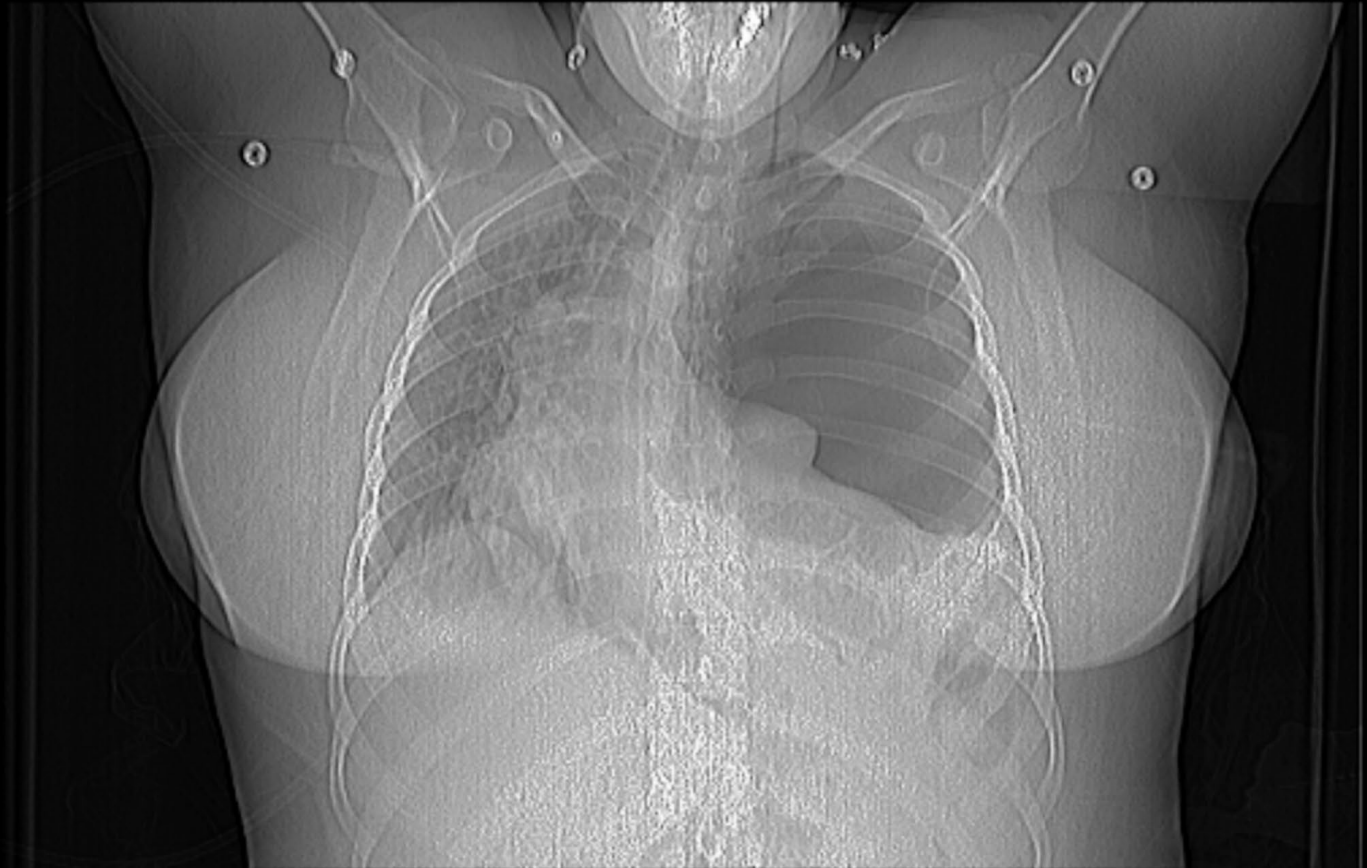
Scout image from the first CT attempt shows missing normal pulmonary pattern on the left with significant mediastinal right shift indicating high tension PTX and the reason of the poor respiratory condition

Pneumonia was considered the most likely diagnosis, though pulmonary embolism (PE) could not be excluded. Following the YEARS algorithm, a D-dimer test was performed which returned a positive result. (Table 1.) A chest CT scan was initiated. However, the initial topographic image revealed a tension pneumothorax (Fig. 1.), prompting the immediate interruption of the examination.

**Fig. 2:**
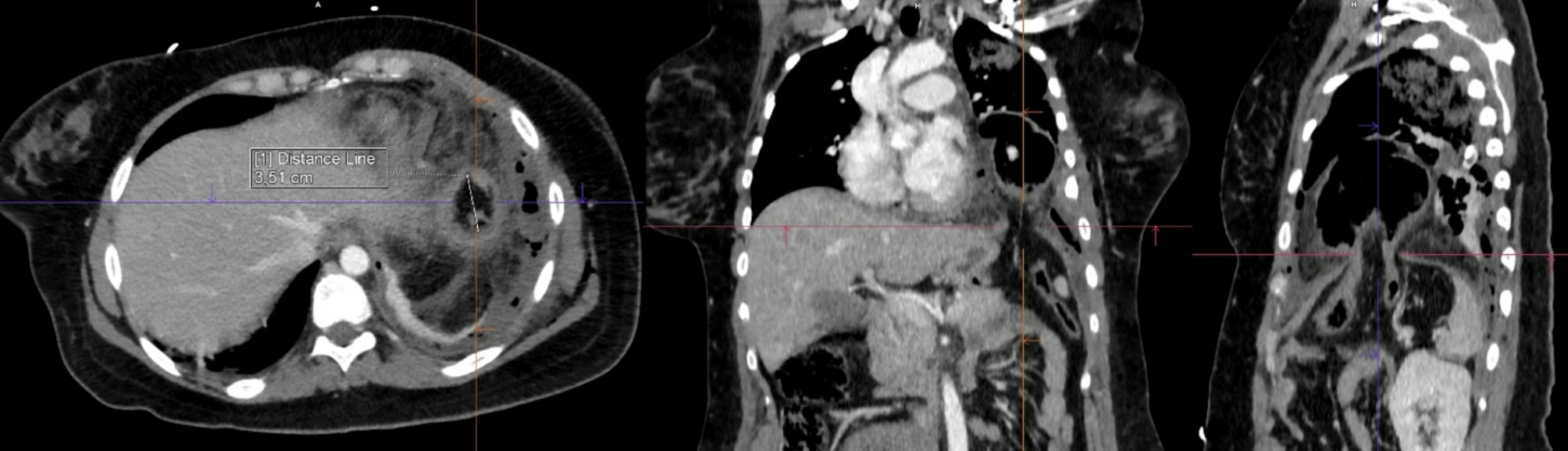
Multiplanar reconstruction of the hernia gate on the left side of the diaphragm. The gate measures 35 mm across. Pleural drain indicated dorsal along the ribs

**Fig. 3:**
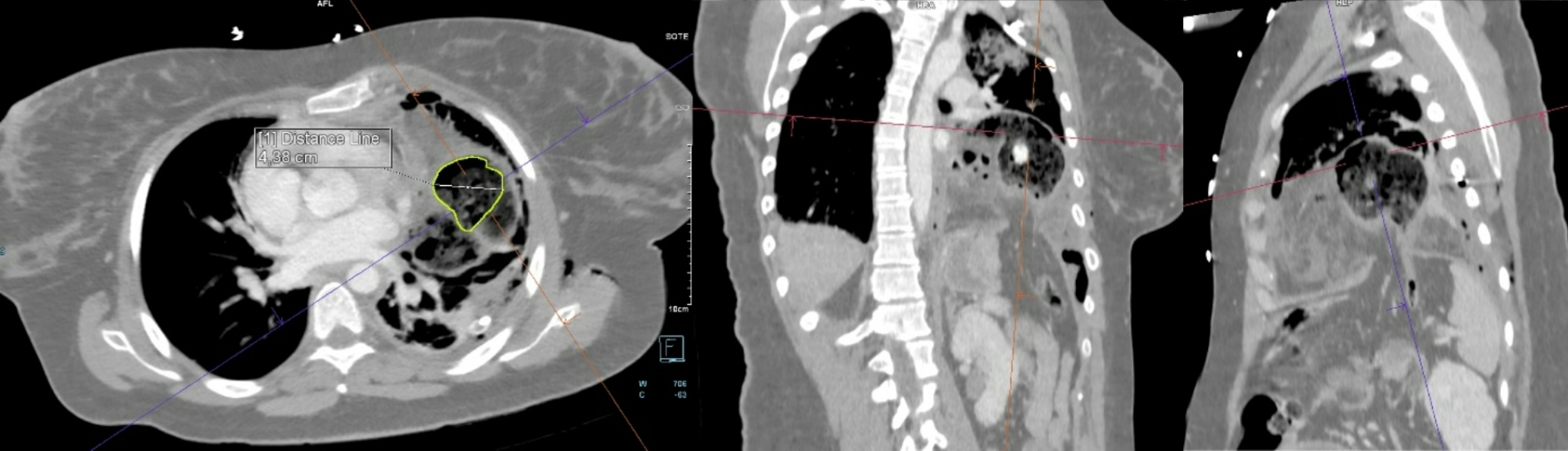
Multiplanar reconstruction of the perforation on the colon, which is highlighted with lime in the (near) axial plane. The perforation measures 44 mm across

**Fig. 4:**
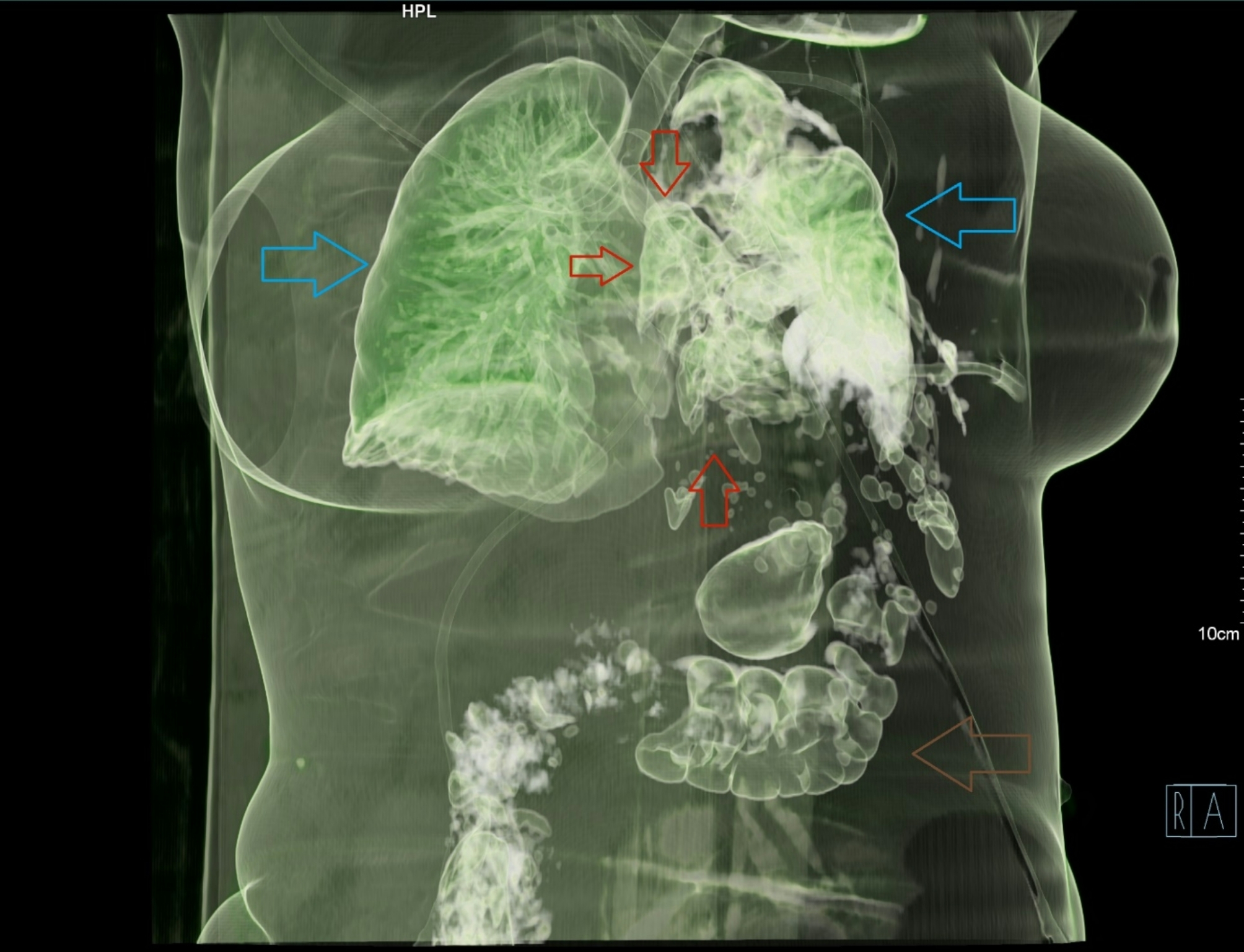
3D reconstruction with shaded surface display (SSD) in a slightly rotated position. The blue arrows point to the normal right lung and the compressed left lung. The brown arrow points to the normal transverse colon. Red arrows point to the gases contained within and around the herniated and perforated part of the colon

**Fig. 5:**
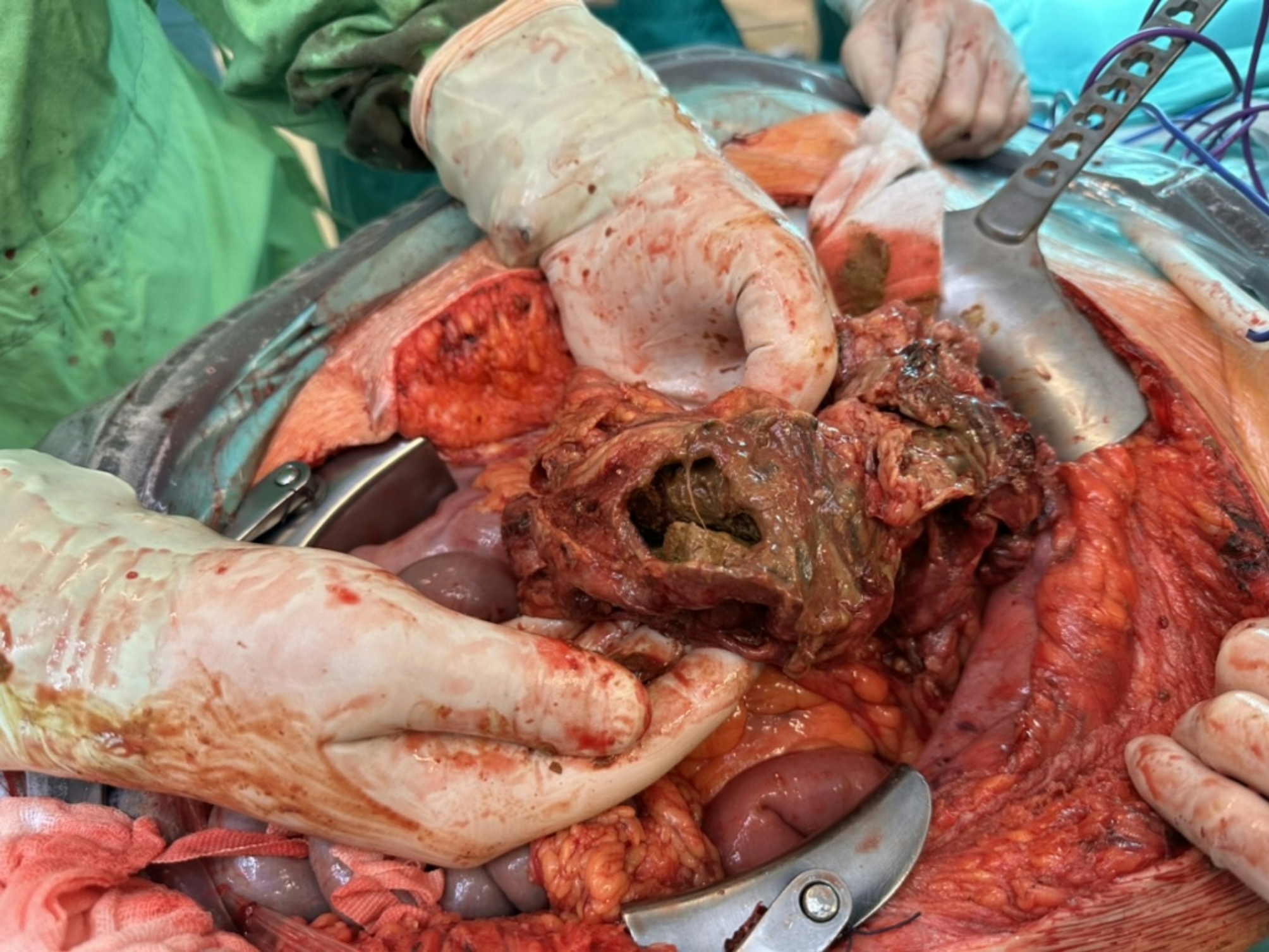
The necrotized and perforated part of the colon

**Fig. 6:**
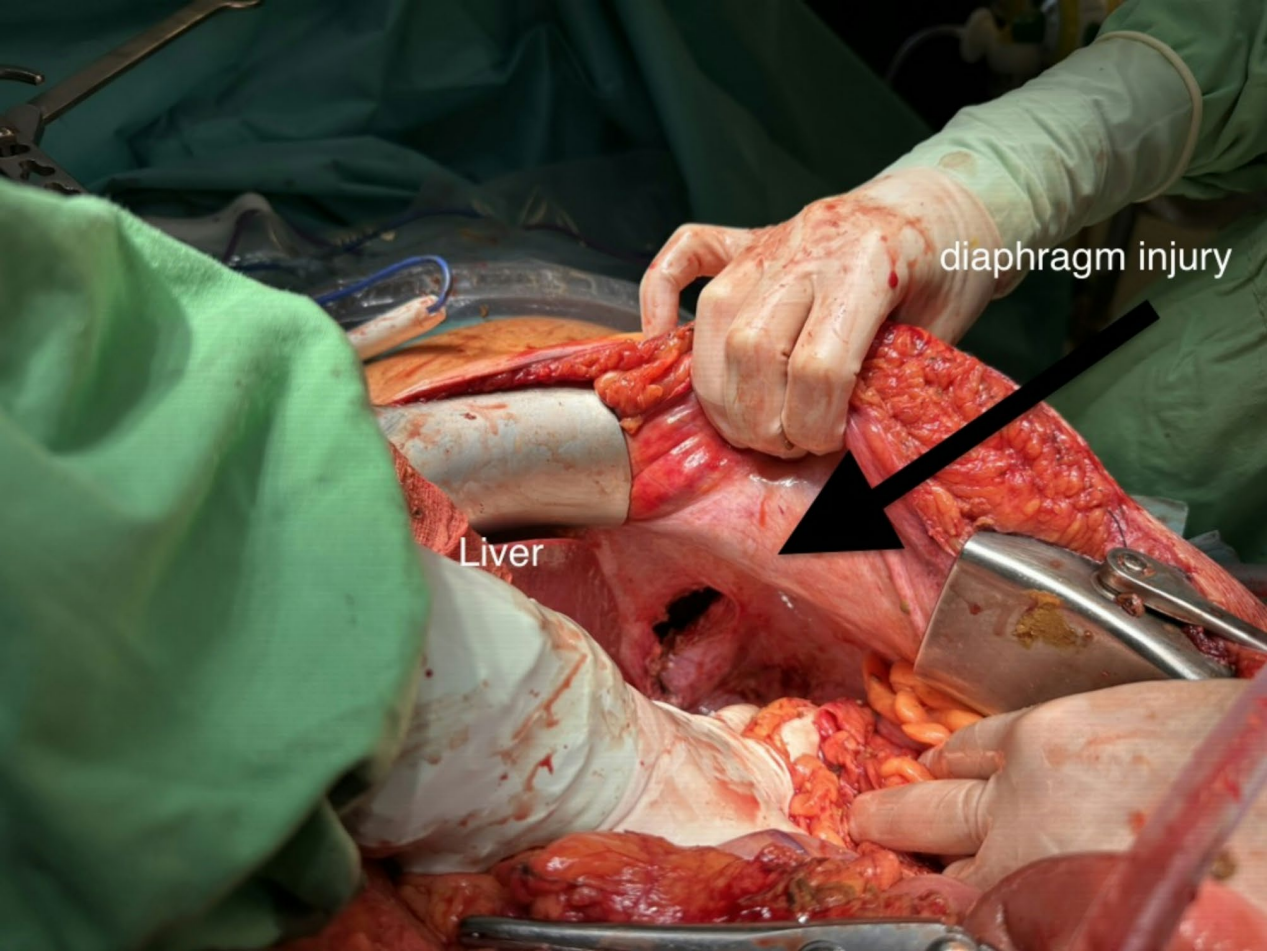
The diaphragmatic injury, after it had to be slightly enlarged to facilitate the withdrawal of the bowel from the thoracic cavity. Original size was 35 mm. (Grade III traumatic diaphragm hernia according to the AAST - American Association for the Surgery of Trauma - classification). It is in the central portion of the diaphragmatic dome, at the junction of the central tendon (centrum tendineum) and the muscular part, slightly posterolateral on the left side and medial to the spleen, which is the most common location for traumatic diaphragmatic ruptures. It has been repaired with simple knotted stitches using size „0” absorbent braided surgical suture. The use of mesh was contraindicated due to the septic condition, but it was also unnecessary, as the surrounding tissues were adequately pliable to allow for primary closure

A chest tube was inserted using a blunt technique, releasing a large volume of air, pus, and fecal matter. A total of 1200 ml of pus mixed with feces was collected, samples were sent for microbiological analysis. The patient’s respiratory function improved slightly, and circulatory parameters remained stable. Following chest tube placement, the CT scan was completed. It revealed a 35 mm diaphragmatic hernia on the left side in an atypical location, with the transverse colon herniated into the thoracic cavity. The herniated colon showed thickened walls, infiltrated fatty tissue, and gas bubbles. A 44 mm colon wall defect, with significant extraluminal feces was identified. Additionally, the left upper lung lobe showed consolidation, while the left lower lobe had extensive atelectasis. A small residual pneumothorax was noted at the apex (Figs. 2, 3, 4, 5 and 6).

An interdisciplinary team—including specialists in emergency care, critical care, anesthesia, surgery (chest and abdominal), obstetrics, and neonatology—was assembled, with the patient actively involved in the decision-making process. After a thorough risk-benefit analysis, surgery was delayed for a few hours. Broad-spectrum antibiotics (piperacillin/tazobactam 4/0.5 g) and glucocorticoids (betamethasone 12 mg) were given, and she was admitted to the critical care unit.

**Fig. 7:**
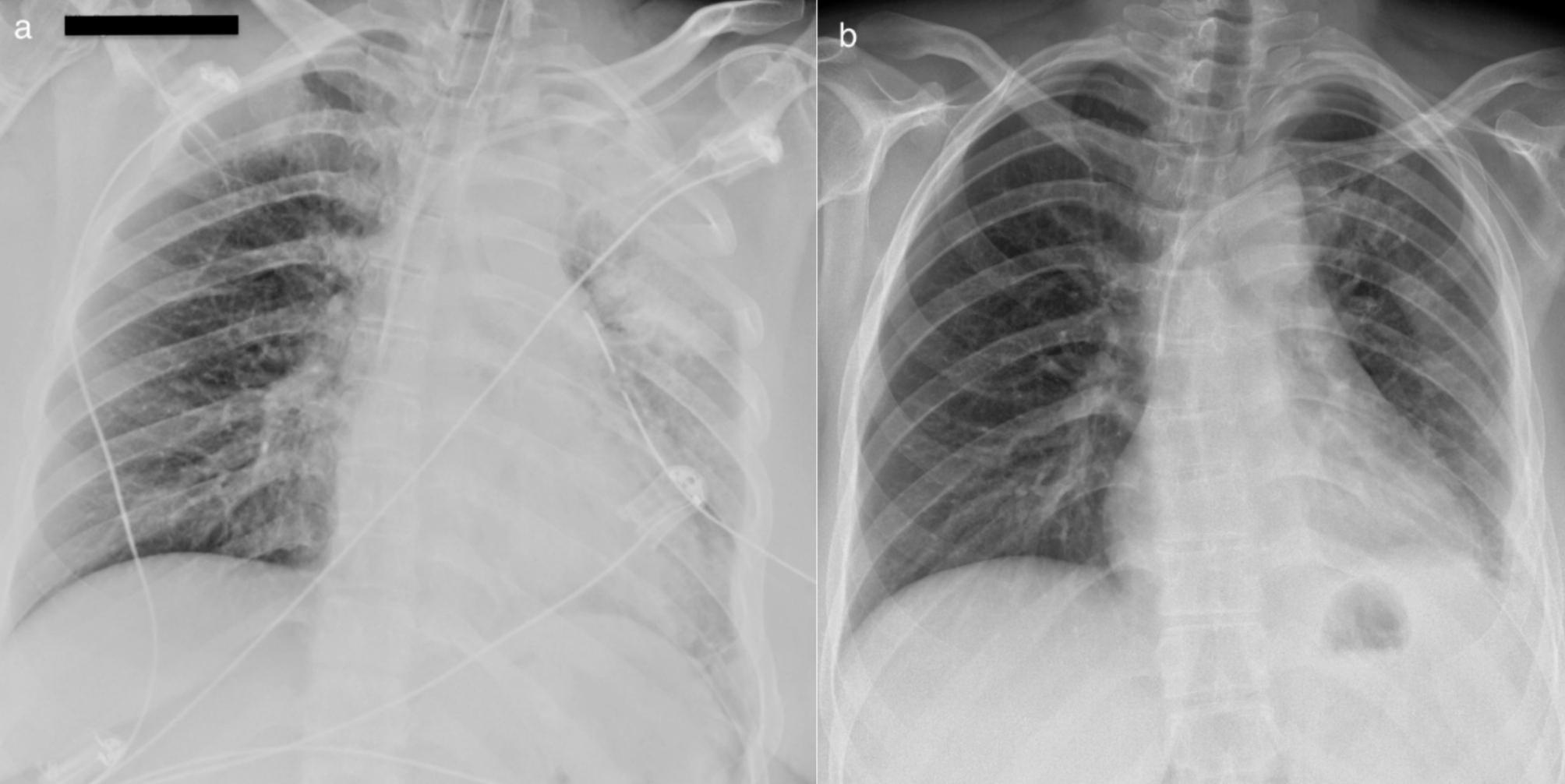
Chest X-ray on the first (**a**) and 14th (**b**) postoperative day

Fetal parameters and umbilical artery flow was normal. With maternal indication (based on the septic state and the expected critical care treatment needs) six hours later, the patient underwent a cesarean section immediately followed by bowel resection, colostomy creation, partial lung resection, and diaphragm repair in the same setting [16]. After 12 days in critical care and a total hospital stay of 38 days, she was discharged in good clinical condition (Fig. 7.). Her enterostomy was successfully closed six months later.

Further investigation revealed a history of thoracic trauma eight years prior, when she was struck by a lorry. At the time, she sustained multiple rib fractures and a left-sided hemopneumothorax, which was managed with a chest drain. However, no imaging or detailed records of the prior injury were available.

Her premature baby, born at 30 + 2 weeks of gestation, weighed 1760 g at delivery, with an Apgar score of 2/7/9. The baby required respiratory support for 30 h and several blood transfusions. He also suffered from bilateral intraventricular hemorrhage with post-hemorrhagic hydrocephalus, which required drainage. Both the mother and baby were discharged on the same day, and follow-up showed that the child’s development was progressing appropriately for his age.

## Discussion

The objective of this report is to present a case of a pregnant women with tension pneumothorax with an unusual origin as late onset symptom and consequence of a traumatic diaphragmatic hernia. MDH is rare and trauma related cases are only a small proportion of the cases [11, 12]. Late presentation is common after traumatic diaphragm injury. Pregnancy may contribute to delayed symptoms after traumatic diaphragm injury or CDH due to increased intra-abdominal pressure and diaphragm changes [2, 17]. 

Diagnosing MDH is challenging due to its variable origin, severity, onset and broad variety of symptoms which can mimic other conditions and physiological changes in pregnancy [6, 11, 12, 17]. Evaluating pregnant patients with dyspnea in the emergency department is challenging, with a focus on promptly identifying and treating life-threatening conditions. In this case pulmonary embolism had to be investigated. Various diagnostic approaches with limited validation exist [18–20]. The pregnancy-adapted YEARS Algorithm was followed, and a chest CT angiogram (CTA) was performed which unexpectedly revealed tension pneumothorax [19]. However, the American Heart Association recommends performing a chest X-ray first to rule out other pathologies first, which, in retrospect, may have been a better initial choice [20]. 

Spontaneous pneumothorax during pregnancy is extremely rare, with fewer than 100 cases reported [21, 22]. In this case, there was no indication of an iatrogenic or a traumatic origin. Initial treatment of tension pneumothorax involves acute decompression, which was performed via chest tube insertion.

Pneumothorax should have been revealed during the initial US examination. The limited evaluability was possibly caused by the PTX. This highlights the importance of a structured approach when using bedside US [23]. 

The subsequent CT scan confirmed a diaphragmatic hernia, a diagnosis often challenging due to its rarity and nonspecific, delayed symptoms. Chest X-ray is the first-line imaging tool, with CT being the gold standard. In pregnancy, ultrasound and MRI are preferred [6, 24]. 

Pneumothorax alongside herniated abdominal organs has been reported in only a few cases [9, 13, 14, 25–28]. On X-ray, a distended stomach or bowel can mimic pneumothorax, risking misdiagnosis and potential iatrogenic perforation during chest drain insertion [28–33]. Using a blunt technique for chest tube insertion is critical to prevent severe complications.

The combination of tension pneumothorax, bowel perforation, and late presentation of trauma-related diaphragmatic hernia during pregnancy is extremely rare. Bernhardt presented a case with small not tension PTX [13]. Hanekamp reported a case with tension PTX eight years after blunt trauma in a 5 month pregnant woman [14]. Lacayo published a case in which tension PTX developed only after delivery [15]. To the best of the authors knowledge, this is only the third reported case. (Additional file 1.)

A multidisciplinary approach is crucial for management, with treatment decisions guided by the severity of the condition, affected organs, and gestational age [11]. 

## Conclusion

Evaluating pregnant patients with shortness of breath in the emergency department is challenging. Identifying a history of thoracic or abdominal trauma is crucial, as this can raise the suspicion of diaphragmatic hernia, which can present with a wide range of symptoms. A structured approach is essential when bedside emergency ultrasound is used. Spontaneous tension pneumothorax in pregnant women is extremely rare and requires cautious management. A multidisciplinary approach is crucial for the successful treatment of MDH.

## Electronic supplementary material

Below is the link to the electronic supplementary material.

Supplementary Material 1

## Data Availability

No datasets were generated or analysed during the current study.

## Abbreviations

ADH: Acquired diaphragmatic hernia
CDH: Congenital diaphragmatic hernia
CRP: C-Reactive protein
CT: Computer tomography
CTA: Computer tomography angiogram
DH: Diaphragmatic hernia
GCS: Glasgow Coma Scale
MDH: Maternal diaphragmatic hernia
PE: Pulmonary embolism
PTX: Pneumothorax
US: Ultrasound
WBC: White blood cell count
