# Supplementary material for: Tension pneumothorax from large bowel herniation and perforation as a late presentation of traumatic diaphragmatic hernia during pregnancy: a case report
Source: Int J Emerg Med. 2025 Mar 3;18:40. doi: 10.1186/s12245-025-00843-1 (PMC11874637; doi:10.1186/s12245-025-00843-1)
Supplement: Supplementary file 1 — Supplementary Material 1 [file 12245_2025_843_MOESM1_ESM.docx]

| author | year | patient’s age | pregnancy | trauma | main symptoms | herniated organs | maternal outcome |
| --- | --- | --- | --- | --- | --- | --- | --- |
| Lacayo L et al. | 1993. | 20 | postpartum (4 hours after caesarean delivery) | 2 years earlier - penetrating | tachycardia, fever, anuria, chest pain, shortness of breath | transverse colon (perforated) | N/A |
| Hanekamp LA et al. | 2006. | 26 | 5 months | 8 years earlier - blunt | shortness of breath, left-sided chest pain, nausea and vomiting | stomach (perforated), colon, spleen | full recovery |
